# High Level of Conservation of Mitochondrial RNA Editing Sites Among Four *Populus* Species

**DOI:** 10.1534/g3.118.200763

**Published:** 2019-01-07

**Authors:** Wolfram Georg Brenner, Malte Mader, Niels Andreas Müller, Hans Hoenicka, Hilke Schroeder, Ingo Zorn, Matthias Fladung, Birgit Kersten

**Affiliations:** Thünen Institute of Forest Genetics, 22927 Grosshansdorf, Germany

**Keywords:** RNA editing, mitochondria, poplar, Populus

## Abstract

RNA editing occurs in the endosymbiont organelles of higher plants as C-to-U conversions of defined nucleotides. The availability of large quantities of RNA sequencing data makes it possible to identify RNA editing sites and to quantify their editing extent. We have investigated RNA editing in 34 protein-coding mitochondrial transcripts of four *Populus* species, a genus noteworthy for its remarkably small number of RNA editing sites compared to other angiosperms. 27 of these transcripts were subject to RNA editing in at least one species. In total, 355 RNA editing sites were identified with high confidence, their editing extents ranging from 10 to 100%. The most heavily edited transcripts were *ccmB* with the highest density of RNA editing sites (53.7 sites / kb) and *ccmFn* with the highest number of sites (39 sites). Most of the editing events are at position 1 or 2 of the codons, usually altering the encoded amino acid, and are highly conserved among the species, also with regard to their editing extent. However, one SNP was found in the newly sequenced and annotated mitochondrial genome of *P. alba* resulting in the loss of an RNA editing site compared to *P. tremula* and *P. davidiana*. This SNP causes a C-to-T transition and an amino acid exchange from Ser to Phe, highlighting the widely discussed role of RNA editing in compensating mutations.

RNA editing is the term for a post-transcriptional process by which the RNA is altered resulting in a sequence deviating from its corresponding genomic template (Benne *et al.* 1986). The alterations encompass insertions, deletions, or chemical modification of single bases. RNA editing sites refer to specific RNA positions affected by RNA editing, and also to the corresponding DNA positions.

In land plants, RNA editing was first discovered in plant mitochondria in 1989 (Covello and Gray 1989; Gualberto *et al.* 1989; Hiesel *et al.* 1989), and somewhat later also in chloroplasts (Hoch *et al.* 1991). In nuclear encoded transcripts, RNA editing was also described, but not extensively analyzed (Meng *et al.* 2010).

In endosymbiont organelles of higher plants, the only RNA editing mechanism is the conversion from C to U by deamination (Takenaka *et al.* 2013), while U-to-C conversion occurs in lycopods, ferns, and hornworts. Insertions and deletions have not been observed in plants, but are present in kinetoplastids, a group of flagellated protists, where the phenomenon was first described (Benne *et al.* 1986).

In some instances, RNA editing occurs with an extent of virtually 100% (*i.e.*, the affected C is edited to U in all transcripts), compensating mutations in the genomic sequence that would otherwise lead to the exchange of highly conserved amino acids in the encoded proteins by restoring the original transcript sequence (Gualberto *et al.* 1989). This view is supported by the circumstance that most RNA editing events occur at position 1 or 2 of a codon, usually altering the encoded amino acid (Takenaka *et al.* 2013). These RNA editing sites are highly conserved across plant species and are efficiently edited as shown recently in a comparison of RNA editing sites in 17 angiosperm species (Edera *et al.* 2018). Another line of evidence for the mutational compensatory mechanism outside of higher plants has been recently provided in dinoflagellates, a photoautotrophic group with extensively edited mRNAs in their organelles and high conservation of editing sites (Klinger *et al.* 2018).

In other instances, RNA editing is a regulated process, meaning that a given editing site may only be edited to an extent of less than 100%, sometimes even to less than 10% (Benne 1989; Simpson and Shaw 1989; Bentolila *et al.* 2013; Takenaka *et al.* 2014). Therefore, RNA editing may serve as a transcriptional control mechanism. This view is supported by the introduction of translational initiation or termination codons by RNA editing (Hoch *et al.* 1991).

Apart from mature mRNA, RNA editing can be found in untranslated regions and introns, where it is in some instances a prerequisite for splicing (Börner *et al.* 1995). It is also thought to be involved in *trans*-splicing (Binder *et al.* 1992). In non-protein-coding RNA species, editing events were identified in tRNAs (Binder *et al.* 1992), whereas editing in rRNAs is rare, if it happens at all (Takenaka *et al.* 2013).

Edited C nucleotides cannot be recognized by a common sequence motif in the vicinity. Thus, editing sites are individually recognized. For a number of editing sites, 20 to 25 bp long *cis* elements have been identified, localized 5′ of the edited C, the crucial residues being 5 to 15 nucleotides upstream (Bock *et al.* 1996; Chaudhuri and Maliga 1996; Verbitskiy *et al.* 2008). In other instances, nucleotides further upstream or downstream of the editing site appear to have influence on editing (Verbitskiy *et al.* 2008). The great variability of both sequence and location of the *cis* elements relative to the edited nucleotide imply that different site-specific *trans* factors individually recognizing single editing sites direct RNA editing (Takenaka *et al.* 2013). The proteins of the PLS-class of pentatricopeptide repeat (PPR) proteins have been identified as the *trans* factors in RNA editing (Kotera *et al.* 2005; Hammani *et al.* 2009; Zehrmann *et al.* 2009; Barkan and Small 2014; Takenaka *et al.* 2014). The PPR proteins are encoded in the nuclear genome, but the translated proteins are almost exclusively targeted to plastids and mitochondria (Colcombet *et al.* 2013). As summarized in several reviews (Barkan and Small 2014; Takenaka *et al.* 2014), the PPR proteins are characterized by a number of consecutive tandem modules, each of which binds to a specific upstream nucleotide (Kindgren *et al.* 2015). PPR proteins may contain a DYW element which is expected to act as the deaminase enzyme (Barkan and Small 2014; Takenaka *et al.* 2014). When a DYW element is missing, a second PPR protein contributing the DYW function may be recruited with the support of the MORF/RIP proteins. Additional proteins unrelated to PPRs are also involved in organellar RNA editing, suggesting that the process is mediated by complex editosoms (Barkan and Small 2014; Takenaka *et al.* 2014).

A straightforward way to detect RNA editing sites is to compare RNAs with their corresponding DNA templates. As an alternative approach to Sanger sequencing of cDNAs (Gualberto *et al.* 1989; Giegé and Brennicke 1999), next-generation sequencing of transcriptomes (RNA-seq) is increasingly being used for the identification of C-to-U RNA editing sites in recent years (Picardi *et al.* 2010; Fang *et al.* 2012; Grimes *et al.* 2014; Wu *et al.* 2015; Sahraeian *et al.* 2017; Edera *et al.* 2018). Although poly(A)^+^ RNA is usually not (rarely) present in the organelles (Schuster and Stern 2009), poly(A)^+^ RNA in combination with oligo-dT priming for reverse transcription was successfully used for assessing RNA editing in many studies (*e.g.*, Picardi *et al.* 2010; Shearman *et al.* 2014). However, quantitative analysis by such an approach should be handled with care (Stone and Storchova 2015).

In this approach, RNA-seq reads are mapped to genomic sequences (ideally of the same genotype) to identify editing sites and to quantify their editing extent. This strategy is challenging because RNA editing site detection can be distorted by genomic reads that might still be present in RNA-seq data and by RNA-seq reads that may originate from nuclear loci in case of dual transcription of homologs (Choi *et al.* 2006) and map unspecific to the mitochondrial genome sequence. Especially the adjustment of mapping parameters is difficult because stringent mapping settings may lead to false negatives, while more relaxed settings may increase the number of false positives (Guo *et al.* 2015; Edera *et al.* 2018). Nevertheless, this strategy allows a transcriptome-wide fast detection of editing sites and has enormous potential to deepen our knowledge of transcriptional processes in plant mitochondria (Edera *et al.* 2018).

This study focused on the identification of RNA editing sites in the coding sequences of mitochondrial genes in four different *Populus* species to deepen our understanding of RNA editing in this genus. Because RNA-seq data are still rare for *Populus*, RNA-seq data sets from different tissues have been used in this study, taking into consideration that tissue-specific RNA editing events cannot be excluded (Picardi *et al.* 2010; Tseng *et al.* 2013; Chen *et al.* 2017; Ichinose and Sugita 2017; Rodrigues *et al.* 2017) which potentially could restrict species comparisons for some editing sites.

## Materials and Methods

### Detection and plotting of RNA editing sites

Detection of RNA editing sites relied on SNP detection comparing sequencing reads of transcriptomic experiments (RNA-seq) with a genomic template. The sequencing runs used for this study (RNA-seq runs downloaded from SRA at NCBI or newly generated runs available at the SRA of NCBI: PRJNA514029) are listed in Table S1. The genomic template was a FASTA file containing all 78 potentially transcribed RNAs including hypothetical genes, rRNAs, and tRNAs derived from the annotated mitochondrial genome of *P. tremula* W52 (Genbank accession KT337313; Kersten *et al.* 2016). The NGS reads were mapped to the set of 78 transcripts using CLC Genomics Workbench (CLC-GWB) Version 11.0 (QIAGEN, Venlo, The Netherlands), which provided all tools mentioned below. The detailed parameters are listed in Table S2. In brief, read data (QC controlled and – if necessary – trimmed using the *Trim Reads* tool) were used as the input for the *Map Reads to Reference* tool. The resulting read mappings were used as the input for the *Local Realignment* tool. The Reads Track output was then used by the *Low Frequency Variant Detection* tool to produce a Variant Track. The SNP tables contained within the Variant Track output files and the detailed mapping coverage reports were exported from CLC-GWB. Both mappings for single reads and mappings combining multiple reads from species, accessions, etc. were carried out this way. At this stage, coverage and count filters were kept deliberately relaxed in order to investigate as much of the dataset as possible. More stringent filtering was applied at later steps of the analysis (see below).

The SNP tables were filtered for C-to-T polymorphisms. These filtered tables were analyzed using a custom R script (File S1) alongside with the mapping coverage report and the FASTA file containing the genomic information in order to produce graphical representations of editing sites, frequency, and coverage. Stringent filtering for coverage, count, and frequency was performed here using the following para-meters: Minimum coverage ≥ 10, Minimum count ≥ 3, Minimum Frequency ≥ 10, Probability ≥ 0.95.

A summarizing table including all RNA editing sites identified in the four species analyzed was generated using a modified version of Variant Tools (File S2). The original version of the software Variant Tools is available on https://github.com/ThuenenFG/varianttools (Schroeder *et al.* 2016).

### Codon position affected by RNA editing and amino acid changes produced by RNA editing

Codon positions affected by RNA editing were identified based on the position of a related RNA editing site in the CDS using simple equations. The following equations are true for the different codon positions: position mod 3 = 1 → codon position 1; position mod 3 = 2 → codon position 2; position mod 3 = 0 → codon position 3.

Codon changes and amino acid changes produced by RNA editing were identified using an in-house Ruby script where the following exceptions from the standard genetic code were considered: UGA→Trp and CGG→Trp (Table S3b).

### Sequencing, assembly and annotation of the complete mitochondrial genome of Populus alba clone Monrepos

Total genomic DNA was isolated from leaves of the male *P. alba* clone Monrepos (original provenance: Germany, Baden-Wuerttemberg) according to a published protocol (Dumolin *et al.* 1995). Genomic library generation and sequencing on the Illumina MiSeq v3 (2x300 bp paired-end reads; 25x coverage) and on the PacBio RS (10x coverage) was done by GATC Biotech AG (Konstanz, Germany).

Initial quality control of the NGS reads was done with FastQC (http://www.bioinformatics.babraham.ac.uk/projects/fastqc/). If not otherwise stated, CLC-GWB (v. 10.0.1; CLC bio, A QIAGEN company; Aarhus, Denmark) was used for data processing. Using the *Trim Reads* tool, all reads were trimmed including adapter trimming, quality trimming (quality limit of 0.01), trimming of ambiguous nucleotides (maximal two nucleotides allowed), trimming of 10 nucleotides at the 5′-end and 1 nucleotide at the 3′-end and removing reads of less than 80 bp in length. All other options were set to default. In total 218,626 contigs of a length of at least 200 bp were generated by *de novo* assembly of all trimmed reads, using the *De novo Assembly* tool. The mapping mode was set to “Map reads back to contigs” (using a length fraction of 0.9 and a similarity fraction of 0.95). All other parameters were set to default. Duplicates and containments (>=98% identity) were removed using Dedupe included in BBMap (https://sourceforge.net/projects/bbmap/). The remaining 207,725 contigs were subjected to the *Join Contigs* tool of the *Genome Finishing Module* (plugin in CLC-GWB). Contig analysis type was set to “use long reads”, where all PacBio subreads were used as long reads. This step was repeated three times. The longest scaffold representing the entire mitochondrial genome was selected from the scaffolds. Overlapping sequence ends were removed from this scaffold and a N-strech inside the sequence was replaced by a related sequence obtained from one of the original MiSeq contigs.

The entire mtDNA sequences of *P. alba* clone Monrepos (838,420 bp; Genbank accession MK034705) was annotated based on the annotations of the mtDNA sequence of *P. tremula* W52 (KT337313; Kersten *et al.* 2016). Briefly, the related GenBank file (KT337313.1) was transferred to a draft SQN-file using the CHLOROBOX-GenBank2Sequin-tool (https://chlorobox.mpimp-golm.mpg.de/GenBank2Sequin.html). This SQN-file was edited using the Sequin tool (v13.05; https://www.ncbi.nlm.nih.gov/Sequin/) by importing the new mtDNA sequence of *P. alba* (clone Monrepos) with “update sequence”. A GenBank file of the *P. alba* mtDNA was exported from Sequin and served as input to create the related circular gene map using the OrganellarGenomeDRAW software (OGDRAW v1.2, https://chlorobox.mpimp-golm.mpg.de/OGDraw.html; Lohse *et al.* 2013).

### Detection of DNA polymorphisms in Populus CDS and analyses of nad6-146 in different individuals

The reference CDS sequences of *P. tremula* W52 (Genbank accession KT337313) were used as query in a BlastN analysis *vs.* the CDS sequences of *P. davidiana* (KY216145.1) and *P. alba* clone Monrepos (MK034705) extracted from the related GB accessions. All SNPs *vs.* the *P. tremula* reference identified in the alignments were listed (Table S4).

### Data availability statement

The authors affirm that all data necessary for confirming the conclusions of this article are represented fully within the article, its tables, figures, and supplemental material deposited at figshare. RNA-seq data of *P. tremula* and *P. trichocarpa* generated in this study are publicly available at the SRA of NCBI (PRJNA514029). The annotated complete mtDNA sequence of *P. alba* clone Monrepos is available at GenBank (MK034705). Supplemental material available at Figshare: https://doi.org/10.25387/g3.7166141.

## Results

### Identification of C-to-U RNA editing sites in mitochondrial CDSs of four Populus species

RNA editing sites (C-to-U) were identified based on mappings of RNA-seq data of *P. tremula*, *P. alba*, *P. davidiana* and *P. trichocarpa* (Table 1; Table S1; newly generated RNA-seq data at the SRA of NCBI: PRJNA514029) to coding sequences (CDSs) of 34 mitochondrial genes previously annotated in *P. tremula* W52 (Genbank accession KT337313; Kersten *et al.* 2016) including putative CDS of *rpl16* and *mttb*, both annotated as potential pseudogenes (CDS sequences of the genes analyzed are given in File S3).

**Table 1:** RNA-seq data sets from four *Populus* species used in the study

Species (Section)	Genotypes	Total number of reads	Total amount of data (Gb)
*P. tremula* (Populus)	W52, W100, Asp201*^a^*	1,763,130,526	178.08
*P. davidiana* (Populus)	Palgong2*^a^*, Seogwang9*^a^*, Seogwang15*^a^*	1,680,315,108	169.71
*P. alba* (Populus)	*P. alba* var. *pyramidalis**^a^* (no genotype information)	362,749,552	54.41
*P. trichocarpa* (Tacamahaca)	Muhle_Larsen, NW7_17C, Weser4, Weser6	1,680,315,108	211.72

a Data downloaded from NCBI (SRA). Details on the data sets are provided in Table S1. RNA-seq data of *P. tremula* (W52 and W100) and *P. trichocarpa* generated in this study are publicly available at the SRA of NCBI (PRJNA514029).

**Figure 1:**
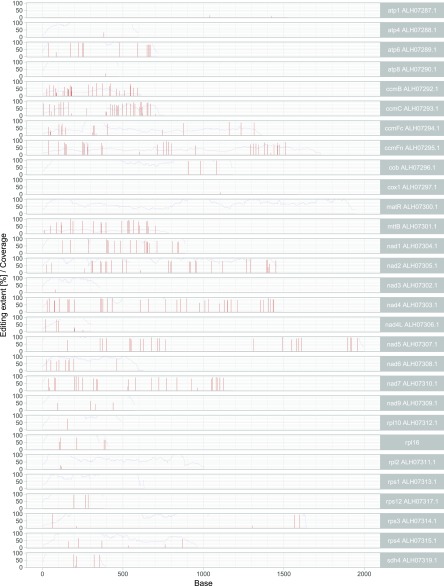
Potential RNA editing sites in 29 mitochondrial CDS of *P. tremula*. In total, 377 potential RNA editing sites identified in combined RNA-seq data sets of three *P. tremula* genotypes (Table 1) were plotted to the nucleotide positions (Base) of the related CDS annotated in *P. tremula* W52 (Genbank accession KT337313; Kersten *et al.* 2016). Bars in red indicate edited bases (editing sites), their height shows the editing extent in percent. Blue lines show the coverage at each base as long as it is 100 or below. All 29 CDS that are potentially affected by RNA editing in at least one of the four *Populus* species investigated are shown in individual rows.

In total, 377 potential RNA editing sites (Table S3a) were identified in the CDS of 29 of the 34 mitochondrial genes analyzed, when considering all editing sites detected in at least one of the four *Populus* species. For all 29 CDS with RNA editing sites, the sites were plotted as red bars to their related nucleotide position as presented for *P. tremula* in Figure 1 (Figure S1 for the other three *Populus* species). The editing extent as given by the height of the red bars was in the range of 10–100%. A value of 10% was set as threshold for the SNP frequency (equivalent to editing extent) when filtering original SNP data according to SNP frequency values. The coverage value at each position is plotted as a blue line allowing to check if there are regions in the CDS escaping RNA editing site detection by an insufficient coverage value (coverage threshold was set to 10 reads in SNP filtering).

**Figure 2:**
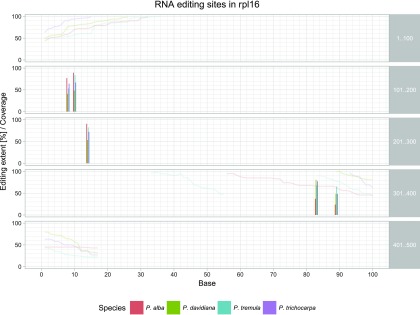
RNA editing in *rpl16* in four *Populus* species. The mitochondrial *rpl16* gene (417 bp) shows five editing sites at positions 108, 110, 214, 383 and 389 bp, which are conserved in the four *Populus* species investigated. Bars indicate edited bases, their height shows the editing extent in percent. Lines show the coverage at each base as long as it is 100 or below. The four *Populus* species are color-coded in both bars and lines.

To make comparisons between species easier, the potential RNA editing sites of all species were plotted together in individual graphical representations of the 29 genes (Figure S2) as shown for *rpl16* as an example in Figure 2. In the CDS of this gene, five RNA editing sites were identified occurring in all four *Populus* species.

The annotation of the complete DNA sequence of the *P. tremula* W52 mitochondrial genome (Genbank accession KT337313; Kersten *et al.* 2016) was checked for overlapping CDS to avoid false-positive/negative detection caused by overlaps. The CDS of *cox3* (246,064 to 246,861 bp) and *sdh4* (246,789 to 247,184 bp) – both annotated in forward direction – show a 72-bp overlap. No potential RNA editing site was identified in the overlapping region of both genes (Table S3a).

The 377 potential RNA editing sites identified in at least one of the four *Populus* species in this study (Table S3a) were compared with RNA editing sites recently identified for *P. tremula* in another study (Edera *et al.* 2018).

All sites identified in only one *Populus* species in our study and not identified by Edera *et al.* (2018), were manually validated in the related mappings. In case of *sdh4* and *rps4*, all sites were validated because nucleotide polymorphisms others than C-to-U were detected in some of the mapped reads. These reads mapped unspecifically to the mitochondrial genome and originated from the nuclear genome as proven by BlastN of related *P. tremula* read sequences *vs.* *P. tremula* scaffolds at PopGenIE (http://popgenie.org/; Sundell *et al.* 2015) and *vs.* nuclear *P. trichocarpa* scaffolds at Phytozome (https://phytozome.jgi.doe.gov/; Tuskan *et al.* 2006). The selected *P. tremula sdh4* reads showed 100% identity to a nuclear *P. tremula* scaffold (Potra000847) and 96% identity to *P. trichocarpa* chromosome 4 in a region where the gene Potri.004g049600 is annotated as “similar to *sdh4*”. BlastN analyses of the selected *P. tremula rps4*-reads provided hits with 99–100% identity to Potra185431, a nuclear *P. tremula* scaffold and with 96% identity to *P. trichocarpa* chromosome 18 in the genic region of Potri.018G031500 annotated as “*rps4*, mitochondrial”. These results indicate dual transcription of mitochondrial genes and their nuclear orthologs in the case of *sdh4* and *rps4* in *P. tremula*. Dual transcription of homologs in the nuclear and mitochondrial genomes has been previously reported for *sdh4* in the *Populus* lineage (Choi *et al.* 2006).

**Figure 3:**
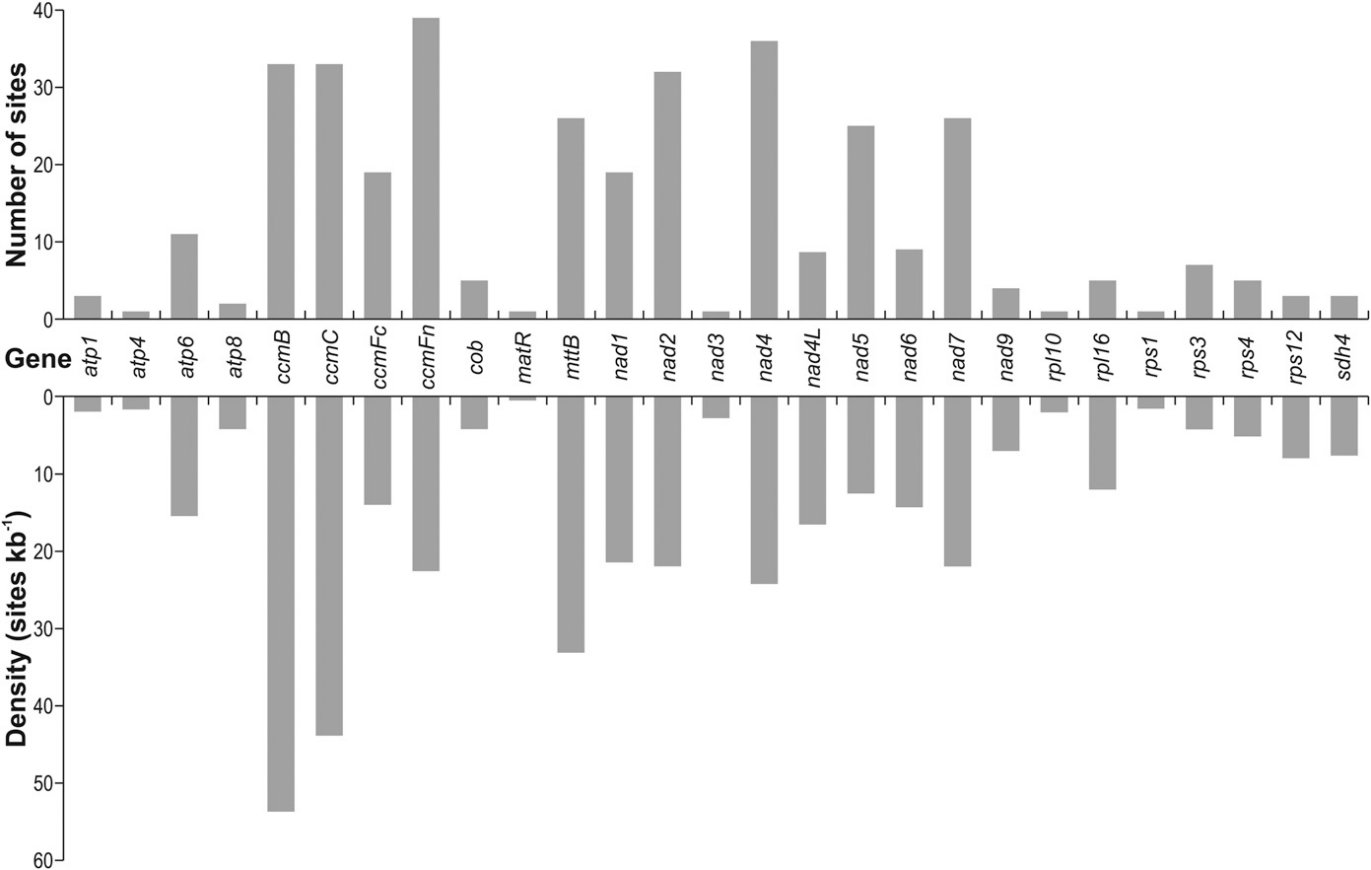
Number (top) and density (bottom) of RNA editing sites in the CDS of 27 mitochondrial protein-coding genes across four *Populus* species. All RNA editing sites detected in at least one of the four *Populus* species investigated are counted. Exact values for the numbers and densities of sites in the related CDS are given in Table S3 (“number and density”).

After this manual validation, 355 RNA editing sites in 27 genes remained (Table S3b). Figure 3 shows the numbers and densities of these RNA editing sites in the related CDS. No editing sites were identified in *atp9*, *cox1*, *cox2*, *cox3*, *rpl2*, *rps7* and *rps14*. In case of *rps7*, the mean coverage of the CDS sequence was below the detection threshold for editing sites in all species except for *P. trichocarpa*.

The highest number of editing sites was identified in the CDS of *ccmFn* (39 sites) and the highest density in the CDS of *ccmB* (53.7 sites/kb; Figure 3).

Most RNA editing sites identified in this study are located at codon position 1 (33%; 118 sites) or 2 (53%; 189 sites). Only 14% of the editing sites (48 sites) are at the third codon position (Table S3b).

### Comparison of the mitochondrial RNA editing sites in the four Populus species

Protein-coding sequences of mitochondrial *Populus* genes were analyzed for DNA polymorphisms to check if there is any overlap with RNA editing site positions identified in this study. Since entire mtDNA sequences with annotated genes were only available for *P. tremula* W52 (Genbank accession KT337313; Kersten *et al.* 2016) and *P. davidiana* Odae19 (KY216145.1; Choi *et al.* 2017), the complete mitochondrial genome sequence of *P. alba* (clone Monrepos) was assembled and annotated in addition (Genbank accession MK034705; Figure S3).

**Figure 4:**
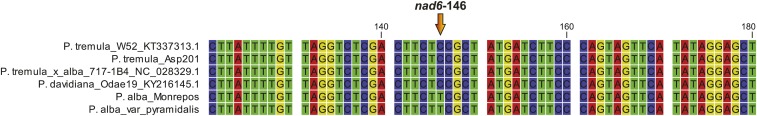
Replacement of an editable cytidine by thymidine at the genomic level in the CDS of *nad6* at position 146 in two *P. alba* genotypes. The *nad6* CDS of *P. alba* clone Monrepos is according to GenBank accession MK034705. The *nad6* CDS of *P. alba* var. pyramidalis was extracted from scaffold GWHAAEP00000188 (105625-106254 bp) of a recent whole genome assembly (Ma *et al.* 2018). The related CDS of other *Populus* species were taken from GenBank accession KY216145.1 (*P. davidiana* Odae19) and KT337313 (*P. tremula* W52). The *nad6* CDS of *P. tremula* Asp201 was extracted from the scaffold Potra197846 (19887-20516 bp) of the *P. tremula* v1.1 whole genome assembly at PopGenIE (http://popgenie.org/; Sundell *et al.* 2015). For *P. trichocarpa*, a related genomic reference sequence is missing. The complete *nad6* alignment is presented in Figure S4.

In total, 16 SNPs were identified in the CDS of the *P. davidiana* and/or the *P. alba* individual when compared with the *P. tremula* individual (Table S3). Only C-to-N or N-to-C SNPs were further considered because only such SNPs may result in a loss or gain of an RNA editing site depending on the location. Only one of these SNPs is located at an RNA editing site, namely a C-to-T SNP identified in *nad6* at position 146 in *P. alba* clone Monrepos (Table S4). The *nad6*-CDS sequences of more *Populus* individuals were compared (Figure 4; Figure S4). In all individuals with a *P. tremula* or *P. davidiana* mitochondrial genome, a C-allele was detected at position *nad6*-146, whereas a T-allele was identified in the two *P. alba* genotypes including a *P. alba* var. pyramidalis individual which was analyzed in a recent whole genome assembly (Ma *et al.* 2018) and which was the source individual of the RNA-seq data used in this study (Table S1). The C-to-T SNP at *nad6*-146 results in a loss of the related RNA editing site via replacement to thymidine in *P. alba*. The SNP is at codon position 2 and results in a codon change (TCC to TTC) and in an amino acid exchange (Ser to Phe; Table S4). RNA editing at C-146 detected in *P. tremula* and *P. davidiana* results in the same amino acid exchange.

For a comparison of the RNA editing sites in the four *Populus* species, 343 sites identified in at least one species were considered, which are covered by at least 10 reads in all four species analyzed (Table S3d). Among these 343 RNA editing sites, 238 sites were identified in all four *Populus* species (Table S3e), indicating that most of the RNA editing sites are highly conserved between the individuals analyzed and probably between the four related *Populus* species. The individual differences at the other RNA editing sites, especially differences observed in the *P. alba* individual compared to the other individuals are expected to be mainly due to a too low coverage at these positions as discussed in more detail below. In case of *P. alba*, only 54 GB of RNA-seq data were available, whereas for the other individuals more than 160 GB of RNA-seq data were included in the study (Table 1).

## Discussion

In our study, 73 new RNA editing sites were detected (355 sites in total) in at least one of the four *Populus* species analyzed. These new sites included 26 sites in *mttb* and 4 sites in *rpl16* (Figure 2) previously annotated as potential pseudogenes because of lacking identification of related start codons (NC_028096.1). No RNA editing sites were reported for these genes by Edera *et al.* (2018) because they did not include these genes in the analysis. The identification of RNA editing sites in *mttb* and *rpl16* in our study suggests both genes are functional genes in *Populus* mitochondria. The expression of mitochondrial-encoded *mttb* has been previously demonstrated on the RNA level in *Nicotiana tabacum* (van der Merwe and Dubery 2007) as well as on the protein level in *Arabidopsis thaliana* (Carrie *et al.* 2016). There are also indications for the expression of *rpl16* in plant mitochondria, where probably a GTG codon acts as translation initiation codon (Bock *et al.* 1994).

Some of the 329 editing sites identified by Edera *et al.* (2018) for *P. tremula* were not identified in our study, among them one editing site in *cox3* and one in *rps14*; both are editing sites with very low RNA editing extent. The detection of RNA editing sites with low editing extent, which is dependent on the threshold set for SNP detection, is difficult and requires sufficient coverage at the related position. Often, a very large amount of RNA-seq data is needed for obtaining enough coverage for mitochondrial genes, as most of the RNA-seq data publicly available are derived from oligo-dT-primed cDNA-libraries and include only a small fraction of mitochondrial RNA molecules (see Introduction). Moreover, contamination of genomic DNA in RNA preparations used for RNA-seq can distort (“dilute”) the values for editing extents.

In general, some differences in identified RNA editing sites and related editing extents are not unexpected between different studies of a species especially if different RNA-seq data sets from different individuals and tissues as well as different methods for the identification of editing sites, especially different mapping parameters are used as in our and Edera’s study (Edera *et al.* 2018). Different strategies have been developed and discussed to improve the detection of RNA editing sites (Guo *et al.* 2015; Stone and Storchova 2015; Edera *et al.* 2018; Wu *et al.* 2018). In mappings of RNA-seq data to CDS, the addition of flanking regions to the CDS may help to increase the read coverage and thus the detection of RNA editing sites at the ends of the CDS (Edera *et al.* 2018).

False-positive RNA editing site detection may also arise from unspecific mapping of nuclear expressed transcripts to mitochondrial reference sequences in cases of dual transcription of nuclear and mitochondrial transcripts as known for *sdh4* in Salicaceae and *Lupinus* (Choi *et al.* 2006; Havird and Sloan 2016) and suggested for *sdh4* and *rps4* in *P. tremula* based on our study. One might circumvent this problem by using genomic sequences of all cellular compartments (nucleus, chloroplast, mitochondrion) of the individual of interest as reference sequences for mappings of RNA-seq data.

False–positive results may also arise when C-to-U RNA editing is “mimicked” by a genomic C-to-T DNA polymorphism at an editable cytidine, which may happen if the genomic reference used for mapping RNA-seq data is from another individual/species than the RNA-seq data.

Substitutions of editable cytidines with thymidines are the main cause of losses of editing sites along angiosperm evolution as shown in 17 genera (Edera *et al.* 2018). The authors expect that consecutive and highly conserved editing sites had been replaced by thymidines (thymidine footprints) as result of retroprocessing, by which edited transcripts are reverse transcribed to cDNA and integrated into the genome by homologous recombination. However, point mutations have also been proposed for the loss of editing sites favored by natural selection (Mower 2008).

Even within one genus, replacements of editable cytidines by thymidine may occur as shown for the loss of the *Populus* RNA editing site *nad6*-146 in two *P. alba* genotypes (Figure 4). Our study indicated that this loss of an RNA editing site could be *P. alba*-specific within the *Populus* genus, however more data are needed to confirm this conclusion. A loss of the *nad6*-146 RNA editing site has also been described in other genera (*Cucumis*, *Malus*, *Arabidopsis* and some Asterids; Edera *et al.* 2018). In general, *Populus* showed the largest number of thymidine footprints and the lowest number of mitochondrial RNA editing sites in the comparison of 17 genera (Edera *et al.* 2018). Early-diverging lineages, such as *Liriodendron* – in contrast – show the highest numbers of editing sites among angiosperms (Richardson *et al.* 2013; Edera *et al.* 2018).

Considering the proportion of RNA editing sites at the different codon positions (33% at position 1; 53% at position 2; 14% at position 3), our results are in agreement with numerous other studies showing that editing sites are predominantly found at non-synonymous positions in protein-coding genes, most frequently at the second position (Giegé and Brennicke 1999; Mulligan *et al.* 2007; Yura and Go 2008; Cuenca *et al.* 2010; Picardi *et al.* 2010; Sloan and Taylor 2010; Edera *et al.* 2018). Recently, it has been shown that conservation levels varied among codon positions across 17 angiosperm genera with lowest conservation at the third positions, as expected for synonymous sites with no obvious impact in the resulting protein (Edera *et al.* 2018).

Among the 355 RNA editing sites identified in this study, 238 sites were identified in all four *Populus* species analyzed (Table S3; “sites_all_species”) indicating that most of the RNA editing sites are highly conserved between the individuals analyzed and probably between the related species. In a recent study in the genus *Leucaena*, 607 conserved RNA editing positions have been identified in the mitochondrial genome when considering all three genome groups in this genus (Kovar *et al.* 2018).

As RNA-seq data sets from various tissues have been used in our study, individual differences due to tissue-specific RNA editing events may not be excluded (Picardi *et al.* 2010; Tseng *et al.* 2013; Chen *et al.* 2017; Ichinose and Sugita 2017; Rodrigues *et al.* 2017). It will be exciting to test in the future whether some of the non-conserved editing sites represent real differences between species and may even have functional implications.

## Conclusions

In this study, the previous finding of Edera *et al.* (2018) that the number of RNA editing sites in poplar mitochondria is the smallest among all angiosperm genera has not only been confirmed, but also expanded from one species to four species within the genus *Populus*. Furthermore, a high level of conservation has been found throughout all poplar species investigated. Interestingly, the loss of an RNA editing site by genomic substitution of an editable cytidine with thymidine was observed in two *P. alba* genotypes. If this finding reflects an ongoing reduction of RNA editing sites within the genus *Populus* cannot be clarified without deeper phylogenetic analyses in the future.

## References

[bib1] Barkan A. Small I., 2014 Pentatricopeptide repeat proteins in plants. Annu. Rev. Plant Biol. 65: 415–442. 10.1146/annurev-arplant-050213-040159 24471833

[bib2] Benne R., 1989 RNA-editing in trypanosome mitochondria. Biochim. Biophys. Acta 1007: 131–139. 10.1016/0167-4781(89)90031-6 2465776

[bib3] Benne R. Van den Burg J. Brakenhoff J. P. Sloof P. Van Boom J. H., 1986 Major transcript of the frameshifted *coxII* gene from trypanosome mitochondria contains four nucleotides that are not encoded in the DNA. Cell 46: 819–826. 10.1016/0092-8674(86)90063-2 3019552

[bib4] Bentolila S. Oh J. Hanson M. R. Bukowski R., 2013 Comprehensive high-resolution analysis of the role of an Arabidopsis gene family in RNA editing. PLoS Genet. 9: e1003584 10.1371/journal.pgen.1003584 23818871 PMC3688494

[bib5] Binder S. Marchfelder A. Brennicke A. Wissinger B., 1992 RNA editing in trans-splicing intron sequences of nad2 mRNAs in *Oenothera* mitochondria. J. Biol. Chem. 267: 7615–7623. 1559998

[bib6] Bock H. Brennicke A. Schuster W., 1994 *Rps3* and *rpl16* genes do not overlap in *Oenothera* mitochondria: GTG as a potential translation initiation codon in plant mitochondria? Plant Mol. Biol. 24: 811–818. 10.1007/BF00029863 8193306

[bib7] Bock R. Hermann M. Kössel H., 1996 *In vivo* dissection of *cis*-acting determinants for plastid RNA editing. EMBO J. 15: 5052–5059. 10.1002/j.1460-2075.1996.tb00885.x 8890178 PMC452244

[bib8] Börner G. V. Mörl M. Wissinger B. Brennicke A. Schmelzer C., 1995 RNA editing of a group II intron in *Oenothera* as a prerequisite for splicing. Mol. Gen. Genet. 246: 739–744. 10.1007/BF00290721 7898443

[bib9] Carrie C. Weißenberger S. Soll J., 2016 Plant mitochondria contain the protein translocase subunits TatB and TatC. J. Cell Sci. 129: 3935–3947. 10.1242/jcs.190975 27609835

[bib10] Chaudhuri S. Maliga P., 1996 Sequences directing C to U editing of the plastid *psbL* mRNA are located within a 22 nucleotide segment spanning the editing site. EMBO J. 15: 5958–5964. 10.1002/j.1460-2075.1996.tb00982.x 8918473 PMC452380

[bib11] Chen T.-C. Liu Y.-C. Wang X. Wu C.-H. Huang C.-H., 2017 Whole plastid transcriptomes reveal abundant RNA editing sites and differential editing status in *Phalaenopsis aphrodite* subsp. *formosana*. Bot. Stud. (Taipei, Taiwan) 58: 38 10.1186/s40529-017-0193-7 PMC5602750 28916985

[bib12] Choi C. Liu Z. Adams K. L., 2006 Evolutionary transfers of mitochondrial genes to the nucleus in the *Populus* lineage and coexpression of nuclear and mitochondrial *Sdh4* genes. New Phytol. 172: 429–439. 10.1111/j.1469-8137.2006.01821.x 17083674

[bib13] Choi M. N. Han M. Lee H. Park H.-S. Kim M.-Y., 2017 The complete mitochondrial genome sequence of *Populus davidiana* Dode. Mitochondrial DNA B Resour. 2: 113–114. 10.1080/23802359.2017.1289346 PMC7800223 33473734

[bib14] Colcombet J. Lopez-Obando M. Heurtevin L. Bernard C. Martin K., 2013 Systematic study of subcellular localization of Arabidopsis PPR proteins confirms a massive targeting to organelles. RNA Biol. 10: 1557–1575. 10.4161/rna.26128 24037373 PMC3858439

[bib15] Covello P. S. Gray M. W., 1989 RNA editing in plant mitochondria. Nature 341: 662–666. 10.1038/341662a0 2552326

[bib16] Cuenca A. Petersen G. Seberg O. Davis J. I. Stevenson D. W., 2010 Are substitution rates and RNA editing correlated? BMC Evol. Biol. 10: 349 10.1186/1471-2148-10-349 21070620 PMC2989974

[bib17] Dumolin S. Demesure B. Petit R. J., 1995 Inheritance of chloroplast and mitochondrial genomes in pedunculate oak investigated with an efficient PCR method. Theor. Appl. Genet. 91: 1253–1256. 10.1007/BF00220937 24170054

[bib18] Edera A. A. Gandini C. L. Sanchez-Puerta M. V., 2018 Towards a comprehensive picture of C-to-U RNA editing sites in angiosperm mitochondria. Plant Mol. Biol. 97: 215–231. 10.1007/s11103-018-0734-9 29761268

[bib19] Fang Y. Wu H. Zhang T. Yang M. Yin Y., 2012 A complete sequence and transcriptomic analyses of date palm (*Phoenix dactylifera L*.) Mitochondrial Genome. PLoS One 7: e37164 10.1371/journal.pone.0037164 22655034 PMC3360038

[bib20] Giegé P. Brennicke A., 1999 RNA editing in *Arabidopsis* mitochondria effects 441 C to U changes in ORFs. Proc. Natl. Acad. Sci. USA 96: 15324–15329. 10.1073/pnas.96.26.15324 10611383 PMC24818

[bib21] Grimes B. T. Sisay A. K. Carroll H. D. Cahoon A. B., 2014 Deep sequencing of the tobacco mitochondrial transcriptome reveals expressed ORFs and numerous editing sites outside coding regions. BMC Genomics 15: 31 10.1186/1471-2164-15-31 24433288 PMC3898247

[bib22] Gualberto J. M. Lamattina L. Bonnard G. Weil J.-H. Grienenberger J.-M., 1989 RNA editing in wheat mitochondria results in the conservation of protein sequences. Nature 341: 660–662. 10.1038/341660a0 2552325

[bib23] Guo W. Grewe F. Mower J. P., 2015 Variable frequency of plastid RNA editing among ferns and repeated loss of Uridine-to-Cytidine editing from vascular plants. PLoS One 10: e0117075 10.1371/journal.pone.0117075 25568947 PMC4287625

[bib24] Hammani K. Okuda K. Tanz S. K. Chateigner-Boutin A.-L. Shikanai T., 2009 A study of new *Arabidopsis* chloroplast RNA editing mutants reveals general features of editing factors and their target sites. Plant Cell 21: 3686–3699. 10.1105/tpc.109.071472 19934379 PMC2798323

[bib25] Havird J. C. Sloan D. B., 2016 The roles of mutation, selection, and expression in determining relative rates of evolution in mitochondrial *vs.* nuclear genomes. Mol. Biol. Evol. 33: 3042–3053. 10.1093/molbev/msw185 27563053 PMC5100045

[bib26] Hiesel R. Wissinger B. Schuster W. Brennicke A., 1989 RNA editing in plant mitochondria. Science 246: 1632–1634. 10.1126/science.2480644 2480644

[bib27] Hoch B. Maier R. M. Appel K. Igloi G. L. Kössel H., 1991 Editing of a chloroplast mRNA by creation of an initiation codon. Nature 353: 178–180. 10.1038/353178a0 1653905

[bib28] Ichinose M. Sugita M., 2017 RNA editing and its molecular mechanism in plant organelles. Genes (Basel) 8: 5 10.3390/genes8010005 PMC5295000 28025543

[bib29] Kersten B. Faivre Rampant P. Mader M. Le Paslier M.-C. Bounon R., 2016 Genome sequences of *Populus tremula* chloroplast and mitochondrion: Implications for holistic poplar breeding. PLoS One 11: e0147209 10.1371/journal.pone.0147209 26800039 PMC4723046

[bib30] Kindgren P. Yap A. Bond C. S. Small I., 2015 Predictable alteration of sequence recognition by RNA editing factors from Arabidopsis. Plant Cell 27: 403–416. 10.1105/tpc.114.134189 25649437 PMC4456925

[bib31] Klinger C. M. Paoli L. Newby R. J. Wang M. Y. Carroll H. D., 2018 Plastid transcript editing across dinoflagellate lineages shows lineage-specific application but conserved trends. Genome Biol. Evol. 10: 1019–1038. 10.1093/gbe/evy057 29617800 PMC5888634

[bib32] Kotera E. Tasaka M. Shikanai T., 2005 A pentatricopeptide repeat protein is essential for RNA editing in chloroplasts. Nature 433: 326–330. 10.1038/nature03229 15662426

[bib33] Kovar L. Nageswara-Rao M. Ortega-Rodriguez S. Dugas D. V. Straub S., 2018 PacBio-based mitochondrial genome assembly of *Leucaena trichandra* (Leguminosae) and an intrageneric assessment of mitochondrial RNA Editing. Genome Biol. Evol. 10: 2501–2517. 10.1093/gbe/evy179 30137422 PMC6161758

[bib34] Lohse M. Drechsel O. Kahlau S. Bock R., 2013 OrganellarGenomeDRAW—a suite of tools for generating physical maps of plastid and mitochondrial genomes and visualizing expression data sets. Nucleic Acids Res. 41: W575–W581. 10.1093/nar/gkt289 23609545 PMC3692101

[bib35] Ma J. Wan D. Duan B. Bai X. Bai Q., 2018 Genome sequence and genetic transformation of a widely distributed and cultivated poplar. Plant Biotechnol. J. 10.1111/pbi.12989 PMC6335071 30044051

[bib36] Meng Y. Chen D. Jin Y. Mao C. Wu P., 2010 RNA editing of nuclear transcripts in *Arabidopsis thaliana*. BMC Genomics 11: S12 10.1186/1471-2164-11-S4-S12 PMC3005917 21143795

[bib37] Mower J. P., 2008 Modeling Sites of RNA Editing as a fifth nucleotide state reveals progressive loss of edited sites from angiosperm mitochondria. Mol. Biol. Evol. 25: 52–61. 10.1093/molbev/msm226 17940211

[bib38] Mulligan R. M. Chang K. L. C. Chou C. C., 2007 Computational analysis of RNA editing sites in plant mitochondrial genomes reveals similar information content and a sporadic distribution of editing sites. Mol. Biol. Evol. 24: 1971–1981. 10.1093/molbev/msm125 17591603

[bib39] Picardi E. Horner D. S. Chiara M. Schiavon R. Valle G., 2010 Large-scale detection and analysis of RNA editing in grape mtDNA by RNA deep-sequencing. Nucleic Acids Res. 38: 4755–4767. 10.1093/nar/gkq202 20385587 PMC2919710

[bib40] Richardson A. O. Rice D. W. Young G. J. Alverson A. J. Palmer J. D., 2013 The “fossilized” mitochondrial genome of *Liriodendron tulipifera*: ancestral gene content and order, ancestral editing sites, and extraordinarily low mutation rate. BMC Biol. 11: 29 10.1186/1741-7007-11-29 23587068 PMC3646698

[bib41] Rodrigues N. F. Fonseca G. C. Kulcheski F. R. Margis R., 2017 Salt stress affects mRNA editing in soybean chloroplasts. Genet. Mol. Biol. 40: 200–208. 10.1590/1678-4685-gmb-2016-0055 28257523 PMC5452132

[bib42] Sahraeian S. M. E. Mohiyuddin M. Sebra R. Tilgner H. Afshar P. T., 2017 Gaining comprehensive biological insight into the transcriptome by performing a broad-spectrum RNA-seq analysis. Nat. Commun. 8: 59 10.1038/s41467-017-00050-4 28680106 PMC5498581

[bib43] Schroeder H. Cronn R. Yanbaev Y. Jennings T. Mader M., 2016 Development of molecular markers for determining continental origin of wood from white oaks (*Quercus* L. sect. *Quercus*). PLoS One 11: e0158221 10.1371/journal.pone.0158221 27352242 PMC4924829

[bib44] Schuster G. Stern D., 2009 RNA polyadenylation and decay in mitochondria and chloroplasts. Prog. Mol. Biol. Transl. Sci. 85: 393–422. 10.1016/S0079-6603(08)00810-6 19215778

[bib45] Shearman J. R. Sangsrakru D. Ruang-Areerate P. Sonthirod C. Uthaipaisanwong P., 2014 Assembly and analysis of a male sterile rubber tree mitochondrial genome reveals DNA rearrangement events and a novel transcript. BMC Plant Biol. 14: 45 10.1186/1471-2229-14-45 24512148 PMC3925788

[bib47] Simpson L. Shaw J., 1989 RNA editing and the mitochondrial cryptogenes of kinetoplastid protozoa. Cell 57: 355–366. 10.1016/0092-8674(89)90911-2 2470509 PMC7133379

[bib48] Sloan D. B. Taylor D. R., 2010 Testing for selection on synonymous sites in plant mitochondrial DNA: The role of codon bias and RNA editing. J. Mol. Evol. 70: 479–491. 10.1007/s00239-010-9346-y 20424833

[bib49] Stone J. D. Storchova H., 2015 The application of RNA-seq to the comprehensive analysis of plant mitochondrial transcriptomes. Mol. Genet. Genomics 290: 1–9. 10.1007/s00438-014-0905-6 25182379

[bib50] Sundell D. Mannapperuma C. Netotea S. Delhomme N. Lin Y.-C., 2015 The Plant Genome Integrative Explorer Resource: PlantGenIE.org. New Phytol. 208: 1149–1156. 10.1111/nph.13557 26192091

[bib51] Takenaka M. Verbitskiy D. Zehrmann A. Härtel B. Bayer-Császár E., 2014 RNA editing in plant mitochondria - Connecting RNA target sequences and acting proteins. Mitochondrion 19: 191–197. 10.1016/j.mito.2014.04.005 24732437

[bib52] Takenaka M. Zehrmann A. Verbitskiy D. Härtel B. Brennicke A., 2013 RNA editing in plants and its evolution. Annu. Rev. Genet. 47: 335–352. 10.1146/annurev-genet-111212-133519 24274753

[bib53] Tseng C.-C. Lee C.-J. Chung Y.-T. Sung T.-Y. Hsieh M.-H., 2013 Differential regulation of Arabidopsis plastid gene expression and RNA editing in non-photosynthetic tissues. Plant Mol. Biol. 82: 375–392. 10.1007/s11103-013-0069-5 23645360

[bib54] Tuskan G. A. DiFazio S. Jansson S. Bohlmann J. Grigoriev I., 2006 The genome of black cottonwood, *Populus trichocarpa* (Torr. & Gray). Science 313: 1596–1604. 10.1126/science.1128691 16973872

[bib55] van der Merwe J. A. Dubery I. A., 2007 Expression of mitochondrial *tatC* in *Nicotiana tabacum* is responsive to benzothiadiazole and salicylic acid. J. Plant Physiol. 164: 1231–1234. 10.1016/j.jplph.2006.11.009 17350139

[bib56] Verbitskiy D. van der Merwe J. A. Zehrmann A. Brennicke A. Takenaka M., 2008 Multiple specificity recognition motifs enhance plant mitochondrial RNA editing *in vitro*. J. Biol. Chem. 283: 24374–24381. 10.1074/jbc.M803292200 18596040 PMC3259818

[bib46] Wu S. Liu W. Aljohi H. A. Alromaih S. A. Alanazi I. O., 2018 REDO: RNA editing detection in plant organelles based on variant calling results. J. Comput. Biol. 25: 509–516. 10.1089/cmb.2017.0214 29641228

[bib57] Wu Z. Stone J. D. Štorchová H. Sloan D. B., 2015 High transcript abundance, RNA editing, and small RNAs in intergenic regions within the massive mitochondrial genome of the angiosperm *Silene noctiflora*. BMC Genomics 16: 938 10.1186/s12864-015-2155-3 26573088 PMC4647634

[bib58] Yura K. Go M., 2008 Correlation between amino acid residues converted by RNA editing and functional residues in protein three-dimensional structures in plant organelles. BMC Plant Biol. 8: 79 10.1186/1471-2229-8-79 18631376 PMC2488346

[bib59] Zehrmann A. Verbitskiy D. van der Merwe J. A. Brennicke A. Takenaka M., 2009 A DYW domain–containing pentatricopeptide repeat protein is required for RNA editing at multiple sites in mitochondria of *Arabidopsis thaliana*. Plant Cell 21: 558–567. 10.1105/tpc.108.064535 19252080 PMC2660620

